# Simulated video-based telehealth training for emergency physicians

**DOI:** 10.3389/fmed.2023.1223048

**Published:** 2023-08-28

**Authors:** Emily M. Hayden, Christopher J. Nash, Susan E. Farrell

**Affiliations:** ^1^Department of Emergency Medicine, Massachusetts General Hospital (MGH), Boston, MA, United States; ^2^Office of Medical Education, Harvard Medical School, Boston, MA, United States

**Keywords:** simulation, telehealth, standardized patients, physicians, assessment

## Abstract

**Introduction:**

Little exists in the literature describing video-based telehealth training, especially for practicing Emergency Physicians.

**Materials and methods:**

This was a retrospective, pre- and post-assessment of physicians’ knowledge and confidence on video-based telehealth after two simulated telehealth encounters. Attending physicians voluntarily participated in Zoom-based trainings and received feedback from the patient actors immediately after each simulation. Post-experience surveys queried participants on the training, aspects of telehealth, and confidence in features of optimal telehealth practice.

**Results:**

The survey had 100% response rate (13/13 physicians). Participants recommended the simulated training experience, mean of 8.38 (SD 1.89; 0 = Not at all likely, 10 = Extremely likely). Pre- and post-response means increased in two questions: “I can describe at least two ways to improve my video-based clinical care”: delta: 1.54, *t*(12) = 3.83, *p* = 0.002, Cohen’s d effect size of 1.06, and “I know when video-based telehealth could be helpful in clinical practice”: delta: 0.99, *t*(12) = 3.09, *p* = 0.009, Cohen’s d effect size of 0.86.

**Conclusion:**

In this pilot, participants viewed telehealth more favorably after the experience and indicated improved confidence in focused telehealth skills. Further study is needed to determine what simulated case content provides the most value for decision-making via telehealth.

## Introduction

Despite the increased use of telehealth, there is a paucity of literature describing training for video-based telehealth, herein “telehealth.” Telehealth instruction frequently focuses on the use of the technology and not provider-patient interactions (1). While the Association of American Medical Colleges Telehealth Competencies exist for trainees and practicing physicians (2, 3), most telehealth training programs described in the literature are designed for medical students (4–7) or residents (8, 9) and not practicing physicians.

Standardized Patient (SP) actors have been used for decades in health professions education to teach and assess learners’ physical examination and communication skills for trainees (4, 5)including a few studies that specifically assesses telehealth-based communication skills (7, 9). To date, there are few descriptions of the use of SPs for training and assessment of practicing physicians’ telehealth skills (10), and only one described its use to orient physicians to a virtual urgent care (11).

Using Kolb’s Four Stages of Learning conceptual framework (12, 13), a two-case, one-hour pilot telehealth training was created for attending-level Emergency Physicians (EPs). During each session, EPs experienced urgent-care cases with trained SPs who subsequently provided feedback on physicians’ communication skills. The cases were written by the authors and included an Ankle Pain and a COVID-19 Infection case (Supplementary Figure 1). The Ankle Pain case was intended to be a straightforward, non-emergent case that did not need imaging, yet would require the providers to depend on the patient to perform diagnostic hands-on examination maneuvers. The COVID case was intended to encourage the clinician to rely on the patient’s observable examination and data (e.g., patient-derived vital signs). These clinical vignettes were created to provide the following learning experiences: (1) feedback on communication skills via telehealth, (2) practice adjusting in-person evaluations to video, and (3) evaluation of diagnostic and referral plans.

This pilot assessed the feasibility of this educational experience, explored participants’ attitudes toward telehealth in general, and the participants’ perceptions of the simulated telehealth experience for practicing focused telehealth skills. Primary outcomes were the participants’ perceived changes in understanding the benefits and limitations of telehealth in their clinical practice. Secondary outcomes included participants’ perceived changes in confidence related to specific aspects of the encounters: determining need for immediate in-person evaluation and the use of patient-generated data.

## Methods

### Setting, population, and human subjects

This was a retrospective, pre- and post-assessment of participants’ knowledge and confidence after a simulated telehealth experience. Eligible participants were attending EPs from an academic, Level 1 trauma center with approximately 110,000 annual patient visits in Eastern Massachusetts. The Institutional Review Board at the study institution approved this pilot study.

The trainings occurred over Zoom (Zoom Video Communications, San Jose, CA) with a group briefing in the main room and individual, 15-min patient encounters in breakout rooms. The SP provided immediate feedback to the physician at the conclusion of each case.

### Outcome measurements

Outcome measures included participants’ self-reported change in knowledge, perceptions, and confidence after experiencing two simulated telehealth video encounters. Collected demographic data included information about participants’ prior experience with telehealth in their clinical practice. Survey data was collected via REDCap (Vanderbilt University, Nashville, TN), using a link emailed to each participant immediately after completing the simulation. Survey responses consisted of Likert scale from 1 = Strongly Disagree to 5 = Strongly Agree (Supplementary Figure 2).

### Data analysis

Data was analyzed using STATA (StataCorp, College Station, Texas, version 17). Descriptive statistics were used for demographic information. Paired T-tests were used to compare the average pre- and post-encounter responses. Effect sizes were calculated by calculating Cohen’s d effect sizes. By convention, Cohen’s d values >0.5 were of medium effect size, and ≥0.8 were of large effect size.

## Results

### Physician demographics

Thirteen EPs participated in the telehealth training pilot. The survey had a 100% response rate. Participant ages ranged from 30 to 70 years. Thirty-nine percent of participants were female. Years of practice as an EP after residency ranged from 0 to 5 to greater than 20. All study participants reported using telehealth prior to this experience.

### Perceptions of the training experience and telehealth

Overall, the participating physicians recommended this training experience with a mean of 8.38 (SD 1.89) on a Likert scale from 0 (Not at all likely) to 10 (Extremely likely). Only one participant (1/13, 7.7%) indicated they would be somewhat unlikely to recommend the experience, indicating a response of 4. The survey assessed mean agreement with the statements outlined in Table 1. Paired *t*-test comparisons of mean agreement pre- and post-learning experience showed increases in two of four questions: 1. “I can describe at least two ways to improve my video-based clinical care”: delta: 1.54, *t*(12) = 3.83, *p* = 0.002, Cohen’s d effect size of 1.06, and 2. “I know when video-based telehealth could be helpful in clinical practice”: delta: 0.99, *t*(12) = 3.09, *p* = 0.009, Cohen’s d effect size of 0.86. Mean increases were also observed in the two questions, “I can describe two benefits of video-based telehealth” and “I can describe two limitations of video-based telehealth,” but these differences did not achieve statistical significance (Table 1).

**Table 1:** Before and after responses of participants.

Survey question	Overall simulation experience					
	Mean before score (SD)		Mean after score (SD)		Difference (95% CI) *p*-value (CI) effect size (Cohen’s d)	
I can describe at least two ways to improve my video-based clinical care.	3.00 (1.2)		4.54 (0.9)		1.54 [0.66, 2.41] *p* = 0.002 *t*(12) = 3.83 1.06	
I can describe two benefits of video-based telehealth.	4.46 (0.78)		4.85 (0.38)		0.77 [−0.08, 0.85] *p* = 0.096 (not significant) *t*(12) = 1.81 0.50	
I can describe two limitations of video-based telehealth.	4.46 (0.66)		4.92 (0.08)		0.78 [−0.01, 0.93] *p* = 0.053 (not significant) *t*(12) = 2.14 0.59	
I know when video-based telehealth could be helpful in clinical practice.	3.85 (1.21)		4.69 (0.48)		0.99 [0.25, 1.44] *p* = 0.009 *t*(12) = 3.09 0.86	

**Survey question**	**Case-specific questions**					
	**COVID case**			**Ankle pain case**		
	**Mean before score (SD)**	**Mean after score (SD)**	**Difference (95% CI) *p*-value (CI) effect size (Cohen’s d)**	**Mean before score (SD)**	**Mean after score (SD)**	**Difference (95% CI) *p*-value (CI) effect size (Cohen’s d)**
I can ascertain that it is safe for a patient to remain at home.	3.77 (0.93)	4.38 (0.51)	0.62 [0.09, 1.14] *p* = 0.025 *t*(12) = 2.55 0.71	3.85 (0.99)	4.54 (0.52)	0.69 [0.18, 1.21] *p* = 0.013 *t*(12) = 2.92 0.81
I can determine if they need to come to the ED now.	4.00 (0.82)	4.46 (0.66)	0.46 [−0.01, 0.93] *p* = 0.053 (not significant) *t*(12) = 2.14 0.59	3.92 (0.86)	4.62 (0.51)	0.69 [0.24, 1.15] *p* = 0.006 *t*(12) = 3.32 0.92
I feel certain in my clinical decision making without the resources I would see in the ED (including vitals, diagnostics, etc.).	2.85 (0.99)	4.08 (0.64)	1.23 [0.62, 1.84] *p* < 0.001 *t*(12) = 4.38 1.22	3.69 (0.85)	4.23 (0.60)	0.54 [0.07, 1.01] *p* = 0.028 *t*(12) = 2.50 0.69
I trust the examination information that I observed, as facilitated by the patient.	3.08 (0.76)	4.08 (0.49)	1.00 [0.40, 1.60] *p* = 0.004 *t*(12) = 3.6 1.00	3.77 (0.93)	4.38 (0.51)	0.62 [0.09, 1.14] *p* = 0.025 *t*(12) = 2.55 0.71

### Confidence with features of telehealth encounter

Survey questions that pertained to the participants’ confidence demonstrated significant differences between the pre- and post-mean ratings for the COVID case on the question “I can determine if they need to come to the ED now.” The effect sizes were highest for the COVID case: “I feel certain in my clinical decision making without the resources I would see in the ED” with a Cohen’s d of 1.2. When considering survey questions with significant difference in pre- and post-means, the Ankle Pain case question with highest effect size was “I can determine if they need to come to the ED now” with a Cohen d of 0.92 (Table 1).

## Discussion

Limited descriptions exist in the literature of the use of simulated telehealth training for practicing physicians, despite a growing need for trained expertise in this segment of healthcare. Given this dearth, this study is novel in both demonstrating training and formative feedback on telehealth skills for practicing physicians within the context of two cases of varied urgency.

Sartori et al. described an SP actor visit embedded in the virtual urgent care physicians’ scheduled shift (11). The physicians were rated using a checklist created from their prior telehealth Objective Structured Clinical Examination for residents (8). While the telehealth skills assessed in the Sartori study were like the ones assessed in this study, the current study differed in that it was a voluntary standalone training program using two cases of different complexity and urgency. Other reported resident- and medical student-level telehealth training using SPs were similar to the Sartori study with the SP sessions being used for assessment after an educational intervention (7, 9), which is different than the current study.

The participants’ baseline scores were already relatively high, which may correlate with all reported having prior experience in telehealth. Prior telehealth experiences were not quantified; however, all EPs in the department have been required to cover telehealth programs. It is possible that the effect sizes may be greater for those practicing physicians who have not previously provided telehealth care.

The effect sizes for all questions were medium to high, demonstrating that this training experience provided an opportunity to improve participants’ telehealth skills as well as their perceptions about telehealth as a care modality. It is known that participating in a simulation case increases a participants’ knowledge (14). Further study is warranted on what aspects of this simulation were most helpful to the participants.

The effect size of the COVID case was large regarding the change in participants’ confidence in clinical decision-making. This is concordant with the intentions for the case—by adding aspects of the patient history of present illness and vital signs in the COVID case to restrain the physician from automatically telling the patient to call 911, yet also creating tension about the safety of the patient remaining at home. The Ankle Pain case was created to be straightforward, and it was not surprising to the authors that the physicians did not find a change in perceived certainty in clinical decision making for this low-acuity case. These findings suggest that providing practice in telehealth cases of potentially unstable patients may better improve physicians’ diagnostic evaluation and decision-making skills during telehealth encounters, while maximizing safe outcomes for telehealth patients.

There were limitations to this pilot study, including the small sample size from a single institution where the use of telehealth is actively being explored. This was a retrospective pre-/post-survey to capture participants’ perceptions prior to the experience which may have contributed recall bias. This study was voluntary, and it unclear how the results may be different if the experience were required. The use of Likert scales may have exaggerated the effect size calculated by the Cohen d statistic, e.g., it is not clear that the difference between a 1–2 response is the same as the difference between a 4–5 response.

In summary, practicing EPs indicated that a simulated telehealth training increased their ability to provide telehealth care and that they viewed telehealth more favorably after a pilot training experience. Future work can explore what aspects of this form of training provide the most value to physicians to optimize patient care via telehealth modalities.

## Data availability statement

The raw data supporting the conclusions of this article will be made available by the authors, without undue reservation.

## Ethics statement

The studies involving human participants were reviewed and approved by Mass General Brigham IRB. Written informed consent for participation was not required for this study in accordance with the national legislation and the institutional requirements.

## Author contributions

EH and SF: study concept and design and acquisition of the data. EH, CN, and SF: analysis and interpretation of the data. EH: drafting of the manuscript. CN and SF: critical revision of the manuscript for important intellectual content. CN: statistical expertise. All authors contributed to the article and approved the submitted version.

## Conflict of interest

The authors declare that the research was conducted in the absence of any commercial or financial relationships that could be construed as a potential conflict of interest.

## Publisher’s note

All claims expressed in this article are solely those of the authors and do not necessarily represent those of their affiliated organizations, or those of the publisher, the editors and the reviewers. Any product that may be evaluated in this article, or claim that may be made by its manufacturer, is not guaranteed or endorsed by the publisher.
