# Supplementary material for: Simulated video-based telehealth training for emergency physicians
Source: Front Med (Lausanne). 2023 Aug 28;10:1223048. doi: 10.3389/fmed.2023.1223048 (PMC10494251; doi:10.3389/fmed.2023.1223048)
Supplement: Supplementary file 1 [file Image_1.pdf]

Supplemental Figure 1. Cases

Ankle Pain

Chief concern: "I hurt my ankle and wonder if I need an x-ray."

HPI:

You are Bobby Smith, a 26-year-old healthy[female/male]. You are calling the Telehealth Line for an urgent visit because you twisted your ankle this morning and you have some questions.

You were rushing to work this morning and while walking downstairs to your kitchen you were pulling a sweater over your head, when you missed the last 2 steps and fell down, twisting your right ankle. You broke your fall by putting your hands out and landed on your butt. Your leg was twisted under you, and it hurt to walk on your right foot after the fall.

You drove to work but your ankle hurt too much to walk and so you drove home early. Note that you were able to use your right foot/leg to still drive home after the injury. You put a bag of frozen peas on your ankle when you got home but now your ankle feels hot and swollen and it hurts when you touch it.
You have 2 main questions:
1. "I'm afraid my ankle is broken. Do I need an x-ray?"
2. "Should I come to the emergency room?"

If asked about discomfort or other symptoms:
You do not have numbness in your foot.
You do not have pain in your calf or your knee.
You had no other injury from the fall.

Past Medical History:

Seasonal allergies

Medications:

none

Over-the-Counter Medications:

vitamins

Allergies:

None

Social History:

You live in a 5th floor walk-up apartment with a roommate and work as a produce manager at a grocery store.
Tobacco use: none
Alcohol use: 2 - 3 drinks on the weekend
Other substance use: marijuana on the weekend

<u>Exam:</u> You appear worried, but in no distress. You can move around and show your ankle if asked. If asked to point or press on your ankle, you point to the most pain just below your outside ankle bone. You do not have other tenderness or pain on other points in your leg/foot/ankle.

Case Objectives:
During the case, the physician will demonstrate that they*:
1. Set up their environment for a successful telehealth visit.
2. Adjusted their in-person communication for a telehealth visit.
3. Adjusted and guided the patient through any physical examination.

*See separate evaluation checklist with more details

COVID Case

Chief concern: "I tested positive for COVID and I want to talk to someone."

<u>HPI:</u>
You are Jaime Hatch, a 76-year-old [female/male] with a history of hypertension. You are calling the Telehealth Line for an urgent visit because you tested positive for COVID yesterday and you have some questions.

You developed cold symptoms 5 days ago: runny nose and congestion, and then developed a cough with clear sputum. You have felt hot and had chills. Yesterday you went to an urgent care clinic and had a rapid COVID test. The clinic called you last night to tell you that your test was positive.

You have not been vaccinated for COVID.
If asked "Why?" you don't have a reason: "Just stupid I guess."
You have not received a flu shot this year: "I get sick with the flu shots. I don't get them."

You have 2 main concerns:
1. Your [wife/husband], who is 72 years old, has several medical problems (high blood pressure, diabetes) and you wonder: "What does this mean for my [wife/husband]?"
2. You have read in the Boston Globe about special antibody therapies for COVID and wonder if you and your wife can get that treatment.

You have 1 main question:
"Should I come to the emergency room?"

If asked about discomfort or other symptoms:
You do not have shortness of breath or difficulty breathing.
You are not sure if your smell or taste is different.
You have no chest pain when you cough.
You have no vomiting and can take your medicine.

You have no diarrhea.
You have no skin rashes.

Past Medical History:
Hypertension
Arthritis in the knees

Medications:
HCTZ 25 mg daily

Over-the-Counter Medications:
Motrin for knee pain, 1 – 2 pills daily

Allergies:
None

Social History:
You live with your [wife/husband] in a retirement complex. Up until the last week, you have continued to socialize with your friends in the game room at the complex, joining them for your daily cribbage game and a drink at happy hour. You do not recall anyone being sick during these encounters.
You have 2 daughters who both live with their families in New York.
Tobacco use: You smoke half a pack per day until you got sick.
Alcohol use: 1 cocktail or beer at happy hour
Illicit substance use: no other drug use

If asked about your [wife/husband]:
"He/she is ok. He/she has a stuffy nose today."

Exam: You appear worried, but in no distress. You can speak in full sentences and do not appear to be short of breath.

Vital Signs:
You checked your blood pressure and heart rate in the recreation room of your retirement complex 1 hour ago. You took your temperature just before you got on this call. You borrowed a finger oxygen sensor when you visited your neighbor in his apartment this morning.
Blood pressure: 158/90
Pulse: 89
Respiratory Rate:
Temperature: 100.8˚F
Oxygen saturation: 97%

Case Objectives:
During the case, the physician will demonstrate that they*:

1. Set up their environment for a successful telehealth visit.
2. Adjusted their in-person communication for a telehealth visit.
3. Adjusted and guided the patient through any physical examination.

*See separate evaluation checklist with more details
