# Supplementary figures and images for: Simulated video-based telehealth training for emergency physicians
Source: Front Med (Lausanne). 2023 Aug 28;10:1223048. doi: 10.3389/fmed.2023.1223048 (PMC10494251; doi:10.3389/fmed.2023.1223048)

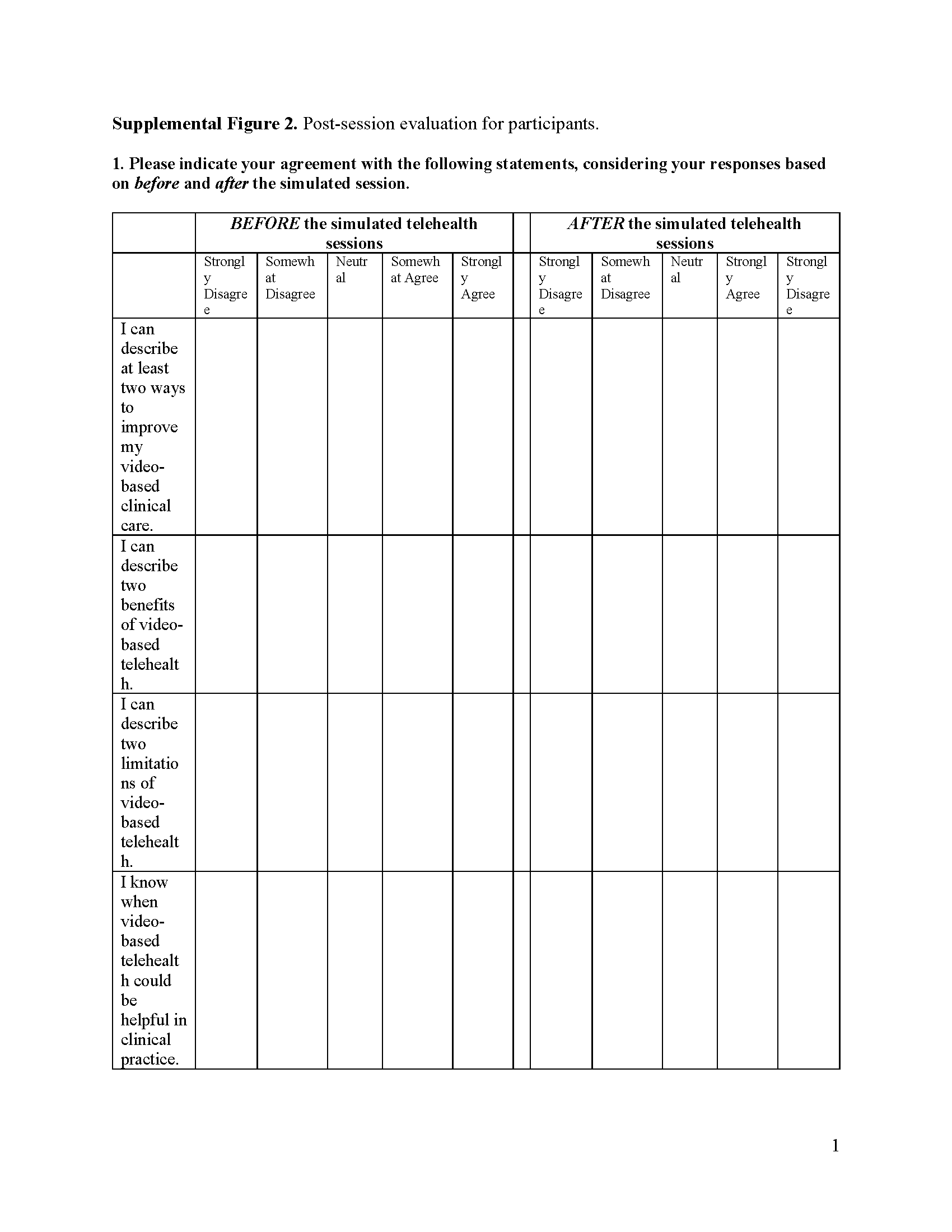

Supplement: Supplementary file 2 [file Image_2.TIFF]
